# Fast Determination of Furocoumarins in Food Supplements Containing *Heracleum sphondylium* L. Using Capillary Electrophoresis

**DOI:** 10.3390/foods14132348

**Published:** 2025-07-02

**Authors:** Eszter Laczkó Zöld, Csenge Kis, Erzsébet Nagy-György, Erzsébet Domokos, Elek Ferencz, Zoltán-István Szabó

**Affiliations:** 1 Department of Pharmacognosy and Phytotherapy, “George Emil Palade” University of Medicine, Pharmacy, Science, and Technology of Targu Mures, 540139 Târgu Mureș, Romania; eszter.laczko@umfst.ro (E.L.Z.); gyorgybobe@gmail.com (E.N.-G.); 2 Department of Physical Chemistry, “George Emil Palade” University of Medicine, Pharmacy, Science, and Technology of Targu Mures, 540139 Târgu Mureș, Romania; csenge.kis@umfst.ro; 3 Faculty of Technical and Human Sciences, Sapientia Hungarian University of Transylvania, 540485 Târgu Mureș, Romania; domokos.erzsebet@ms.sapientia.ro; 4 Legal Medicine Service, Emergency County Hospital Miercurea Ciuc, 530173 Miercurea Ciuc, Romania; elekferencz@yahoo.com; 5 Department of Industrial Pharmacy and Pharmaceutical Management, “George Emil Palade” University of Medicine, Pharmacy, Science, and Technology of Targu Mures, 540139 Târgu Mureș, Romania; 6 Sz-Imfidum Ltd., 525401 Lunga, Romania

**Keywords:** *Hercaleum sphondylium*, hogweed, furocoumarin, electrophoresis, MEKC, sodium cholate, food supplements

## Abstract

*Hercaleum sphondylium* L., commonly known as hogweed, is a plant species that has been employed as an ingredient in food supplements aimed at enhancing reproductive organ functionality, restoring hormonal equilibrium, and producing an aphrodisiac effect. Importantly, the European Food Safety Authority (EFSA) has designated it as a “substance of possible concern for human health” when found in food and food supplements, as detailed in the EFSA compendium of botanicals. Given the potential health consequences associated with the ingestion of furocoumarin-containing plants, the primary objective of this study was to develop a straightforward and rapid method for screening various furocoumarins (bergapten, isobergapten, isopimpinellin, imperatorin, and xanthotoxin) that are found in hogweed plant products and hogweed-derived food supplements. A novel ultrafast micellar electrokinetic chromatographic method was established, achieving analysis durations of less than 3 min for the complete separation of the analytes. This method is additionally characterized by its simplicity, allowing for the analysis of samples following a rapid extraction procedure and dilution, without necessitating extra cleanup steps. The investigation of ten food supplements indicated that seven products contained no detectable levels of furocoumarins, one product presented levels close to the harmless threshold, and two products exhibited concentrations significantly exceeding this threshold. The results of this study illustrate the potential of micellar electrokinetic chromatography as a feasible alternative technique for the analysis of furocoumarins in herbal products and food supplements.

## 1. Introduction

Furocoumarins (or furanocoumarins) represent a class of secondary plant metabolites characterized by an aromatic, tricyclic chemical structure, in which a furan ring is fused to a coumarin. Plants synthesize these compounds in response to stressors such as pest attacks, and they are particularly prevalent in the Apiaceae, Fabaceae, and Rutaceae families [1]. Two types of isomers are distinguished based on the position of the furan ring, namely, angular and linear isomers. Angular isomers, also referred to as “angelicin-type” isomers, and linear isomers, otherwise known as “psoralen-type” isomers, are distinguished by the configuration of their respective rings, specifically the orientation of the furan ring [2]. Several of these substances have been demonstrated to induce phototoxic reactions and can also interfere with metabolic processes [2,3,4]. These compounds have been shown to affect the metabolism of pharmaceuticals through various mechanisms. A multitude of food–drug and herb–drug interactions have been attributed to the induction or inhibition of cytochrome P450 enzymes by linear furocoumarins [4,5]. Chronic administration of psoralen may increase the activity of hepatic drug-metabolizing enzymes such as cytochrome P-450, which may influence the biotransformation rates of co-administered drugs [6]. Furthermore, exposure to plant tissues containing furocoumarins in conjunction with UV exposure can result in phytophotodermatitis, a condition characterized by the formation of blisters [2,7]. Furocoumarins, particularly psoralens, are utilized in phototherapy for the treatment of various dermatological conditions. However, these compounds have the potential to increase the risk of developing skin cancer, a consequence of their ability to form DNA crosslinks when exposed to ultraviolet (UV) radiation [2,8,9]. Recent cohort studies have indicated a positive correlation between the intake of furocoumarins and an elevated risk of developing skin cancer, particularly basal cell carcinoma [10].

Members of the Apiaceae family are typically characterized by a high concentration of coumarins, with the genus Heracleum comprising a substantial quantity of linear and angular furocoumarins [11]. Hogweed (*Heracleum sphondylium* L.) [12] has gained popularity in recent decades. In Romania, it is commonly utilized for self-medication purposes, particularly to address concerns related to potency and fertility, and has gained popularity in the past decades. According to Abbet et al., the young stems and seeds of hogweed are utilized in cooked and salad meals in Canton Valais, Switzerland. Its use in ethnomedicine for treating bronchitis is also documented [13]. Contemporary pharmacological data are scarce. Rat models are being used to assess the vasorelaxant properties of a dichlorometane-obtained extract [14]. The antibacterial and antifungal effects, as well as the antioxidant activity, of several extracts have been explored [15,16].

A multitude of food supplements claiming to possess aphrodisiac properties [17,18] are widely available on the Romanian market. These products target various health complaints associated with hormonal imbalances and enhance reproductive organ function [17,18,19,20,21]. In addition to these reported benefits, *Heracleum sphondylium*-based preparations have been documented to possess immunostimulant, hypotensive, and overall tonic properties that are comparable to those of ginseng [22,23]. There are several forms of *Heracleum sphondylium*-based food supplements, including tinctures, pills, and capsules. These supplements may contain an aqueous or hydroethanolic plant extract, a powdered herbal product (root, aerial parts, or seeds), or a combination of the two. It is imperative to note that these food supplements are sold without the extracts they contain being standardized or checked for their furocoumarin content.

Although *Heracleum sphondylium*, in conjunction with *H. mantegazzianum*, is incorporated within the European Food Safety Authority (EFSA) compendium of botanicals that are reported to contain substances of probable concern for human health when utilized in food and food supplements [24], *Heracleum sphondylium*-based food supplements are commercially available on an international scale through online retailers [25,26]. It is important to note that the EFSA compendium is a preliminary list that does not have the force of law. However, in the case of both species, furocoumarins are mentioned as chemicals of concern. According to the aforementioned scientific guidelines, the daily intake of total furocoumarins in an herbal medicinal preparation that is equal to or below 15 µg is not considered to pose an unacceptable risk to consumers based on the thresholds for toxicological concern [27]. Similarly, the daily intake of 1.5 mg of furocoumarins through herbal medicinal products is not considered to contribute significantly to the overall risk, according to the same set of guidelines, based on the average dietary exposure to furocoumarins [27].

In light of the potential health implications associated with the consumption of furocoumarin-containing plants, implementing stringent quality control measures in the production of food supplements that incorporate these substances is imperative. This necessitates the development of a suitable analytical method that can effectively assist producers in rigorously testing raw materials and extracts to ensure their safety and purity.

Several analytical approaches have been developed for quantifying furocoumarins contained in *Heracleum* species, including *H. sphondylium* [28,29,30]. Ušjak L et al. [29] analyzed the dry dichloromethane (CH_2_Cl_2_) extract of roots and the crystalline precipitate of the CH_2_Cl_2_ extract from the fruits of *H. sphondylium* by liquid chromatography–mass spectrometry (LC-MS). The dominant furocoumarin in the root extract was pimpinellin (266 mg/g extract), followed by isopimpinellin (119 mg/g extract), as well as sphondin (62 mg/g extract), isobergapten (58 mg/g extract), and bergapten (55 mg/g extract) in similar quantities [29]. In the crystalline precipitate of the CH_2_Cl_2_ extract of the fruits, bergapten (70 mg/g precipitate), byankagelicol (68 mg/g precipitate), and heraclenin (44 mg/g precipitate) are dominant [29]. In a recent study, three different subspecies of *Heracleum sphondylium* were investigated for their coumarin content [30] by high-performance liquid chromatography with ultraviolet detection (HPLC-UV). Widely varying amounts of xanthotoxin (0.03–0.05%), angelicin (0.02–0.03%), bergapten (0.001–0.49%), and imperatorin (0.02–0.14%) were detected in the underground parts and aerial parts of *Heracleum* taxa growing in Turkey [30].

Capillary electrophoresis (CE) has emerged as a crucial technique in phytochemical analysis, ranking as the third most widely used method for studying natural products, after high-performance liquid chromatography (HPLC) and gas chromatography (GC) [31]. CE is also often used in the analysis of foods and food supplements [32,33], including the quantification of food colorants and different types of adulterants [33,34,35]. The advantages of CE include low reagent and sample consumption, low running costs, high separation efficiency, rapid method development, and the versatility of changing between various application modes, such as capillary zone electrophoresis (CZE), micellar electrokinetic chromatography (MEKC), and capillary electrochromatography (CEC). Due to its versatility and eco-friendliness, it has been widely applied to phytochemical analysis, as highlighted by numerous recent review articles [36,37,38,39].

Analyses of some of the studied furocoumarins by CE have been reported previously. Wu et al. [40] reported an MEKC method, using 18 mM borate, 12 mM phosphate, and 50 mM sodium dodecyl sulfate (SDS) (pH 9.2) containing 20% methanol as the background electrolyte (BGE) for the separation of five coumarins (xanthotoxin, isopimpinellin, bergapten, imperatorin, and osthol) in Cnidii fructus. The analysis time in this case was around 25 min.

In a 2018 study, Dressler et al. [41] developed an MEKC method for the simultaneous separation of coumarins, including furocoumarins. In the study, six coumarins (coumarin, scoparone, isoscopoletin, esculin, esculetin, and umbelliferone) and six furocoumarins (xanthotoxin, byakangelicin, isopimpinellin, bergapten, phellopterin, and xanthotoxol) were separated simultaneously using sodium cholate as a pseudostationary phase and methanol as an organic modifier. Under these conditions, a selective, reproducible, and high-resolution separation was achieved, albeit at the expense of a 30 min run time.

Zhang et al. [42] reported a dual cyclodextrin (CD)-based BGE for the determination of six furocoumarins, psoralen, isopsoralen, imperatorin, isoimperatorin, phellopterin, and cnidillin, in *Angelica dehurica radix* and its formulations. The analysis time in this case was less than 7 min.

**Figure 1:**
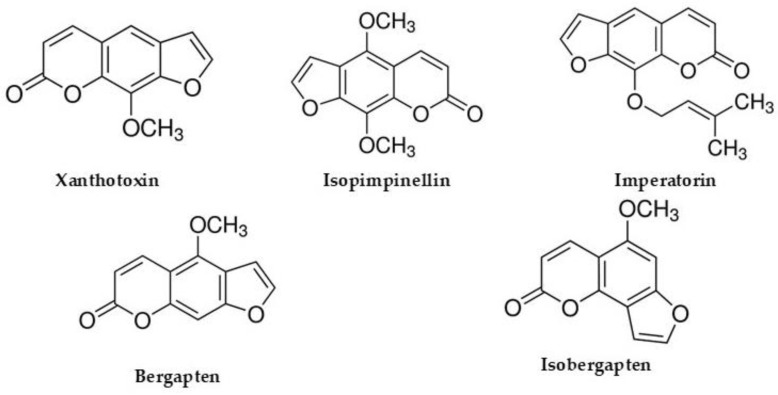
Chemical structures of the furocoumarins used in this study.

In this study, our goal was to develop a rapid and simple CE method for the screening of furocoumarins, including bergapten, isobergapten, isopimpinellin, imperatorin, and xanthotoxin (Figure 1), that are present in *Heracleum sphondylium* herbal products and *Heracleum sphondylium*-based food supplements.

## 2. Materials and Methods

### 2.1. Plant Material

*Heracleum sphondylium* plants were collected by Eszter Laczkó Zöld and Erzsébet Domokos in the Sovata area (Romania, N46° 39.010′ E24° 58.603′. Voucher number: FS/1607/2022; samples 1–6) on 16th of July 2022 during the flowering period and on 7th of August 2022 in Simeria (Romania, latitude 45.856100, longitude 23.01993. Voucher number: FS/0708/2022; sample 7). The plants were identified by Erzsébet Domokos from Sapientia Hungarian University of Transylvania, Faculty of Technical and Human Sciences, Department of Horticulture, Târgu Mureş/Corunca, Romania. The dried parts of the plant were divided into groups: root (sample 1), stem base with root remnants (sample 2), thick stems (sample 3), leaves (sample 4 and sample 7), thin stems (sample 5), and green fruits (sample 6). Voucher specimens of all samples are deposited at the “George Emil Palade” University of Medicine, Pharmacy, Science and Technology of Târgu Mureş, Department of Pharmacognosy (Romania).

### 2.2. Chemicals and Food Supplements

Isopimpinellin (CAS 482-27-9; purity: 99.76%), imperatorin (CAS 482-44-0; purity: 99.86%), isobergapten (CAS 482-48-4; purity: 100%), and xanthotoxin (CAS 298-81-7; purity: 100%) were purchased from PhytoLab GmbH & Co. (Vestenbergsgreuth, Germany), while bergapten (CAS 484-20-8; purity: 98.81%) was acquired from Sigma Aldrich Chemicals Co. (St. Louis, MO, USA). Methanol (MeOH), ethanol (EtOH), and acetonitrile (ACN) of HPLC grade were supplied by Merck (Germany), and dichloromethane (CH_2_Cl_2_) of HPLC grade was purchased from Sigma Aldrich Chemicals Co. (St. Louis, MO, USA).

Sodium tetraborate octahydrate (Na_2_[B_4_O_5_(OH)_4_] ·8H_2_O), NaOH, sodium deoxycholate (SDC), and sodium dodecyl sulfate (SDS) were from Merck (Darmstadt, Germany). Ultrapure water, prepared using a Barnstead Nanopure Diamond water purification system (Boston, MA, USA), was used throughout this study.

CDs used as BGE additives were obtained from Cyclolab (Budapest, Hungary): sulfobutylether-β-cyclodextrin (SBE-β-CD, average degree of substitution (DS)~6), and sulfated-β-cyclodextrin (S-β-CD, DS~13).

Food supplements (samples 8–17) (tinctures, capsules, tablets) labeled as containing *Heracleum sphondylium* were purchased from local pharmacies, drugstores, and online stores in Romania. They were stored in a dark place at room temperature before analysis and were analyzed before their expiration dates (the details of the products are presented in Table S1/Supplementary Material).

### 2.3. Preparation of Analyte Solutions and Herbal Extracts

#### 2.3.1. Furocoumarin Analyte Solutions

Stock solutions of each analyte were prepared in MeOH at a concentration of 1 mg/mL. Additional solutions were prepared by diluting the stock solutions with ultrapure water to obtain the required concentrations. The analyte solution for method development contained all components at approximately 30 µg/mL concentration, except for isobergapten, which was at approximately 10 μg/mL.

#### 2.3.2. Extraction with EtOH

*Heracleum sphondylium* samples were air-dried and stored in a dark location at room temperature prior to analysis. Finely powdered samples were extracted in a 1:10 proportion (*w*/*v*) with 30%, 50%, or 80% EtOH using ultrasound extraction at 20 °C for 20 min. Extracts were centrifuged, and the supernatant was analyzed. Before injection, an adequate volume (2 mL) was passed through a PTFE 0.22 μm filter, and the first 1 mL was discarded. Each extract was prepared in triplicate and stored at 4 °C in the refrigerator until analysis.

### 2.4. Sample Preparation from Food Supplements

Ten capsules from a single bottle were opened, emptied, and mixed. Ten tablets were grounded to a fine powder. Then, 1 g of the samples thus prepared was extracted in a 1:10 proportion (*w*/*v*) with 80% EtOH by ultrasound extraction at 20 °C for 20 min. Extracts were centrifuged, and the supernatant was analyzed. Before injection, an adequate volume (2 mL) was passed through a PTFE 0.22 μm filter, and the first 1 mL was discarded. All samples were stored in the refrigerator at 4 °C until analysis.

Food supplements marketed as tinctures were diluted tenfold with water before testing. The diluted tinctures were also filtered through a PTFE 0.22 μm filter before injection.

### 2.5. Capillary Electrophoretic Conditions

Electrophoretic experiments were performed using an Agilent G1600 CE (Agilent Technologies, Waldbronn, Germany), connected to ChemStation software (Rev. B 04.03) for data processing and interpretation. The equipment features a photodiode array detector, enabling the detection at multiple wavelengths, as well as recording the UV spectrum of the analytes. Throughout the study, an untreated fused-silica extended light path (bubble cell) capillary (50 µm I.D., optical path length = 150 µm, bubble factor = 3, 360 µm O.D., 38.5 cm total length) with a polyimide protective layer (Agilent, Germany) was used. Before its first use, a new capillary was conditioned by flushing with 1 M NaOH for 15 min, followed by 0.1 M NaOH for 15 min, water for 10 min, and buffer exchange (BGE) for 5 min. The same preconditioning steps were performed at the beginning of each working day. Between each run, the capillary was preconditioned using the following procedure: 0.1 M NaOH for 2 min, water for 1 min, and BGE for 2 min. The detection was performed at three different wavelengths: 210 nm, 268 nm, and 315 nm. During the runs, the injection was performed on the short end of the capillary (effective capillary length: 8.5 cm) by applying a pressure of −30 mbar for 2 s. During the electrophoretic runs, the separation voltage was set to 30 kV. Unless stated otherwise, the capillary temperature was maintained at 25 °C.

During method development, experiments were conducted using sodium tetraborate-based background electrolytes (BGEs) at varying concentrations (10 mM, 20 mM, and 30 mM). CDs were tested as BGE additives at three different concentrations (2.5 mM, 7.5 mM, and 10 mM), while surfactants were tested at five different concentration levels ranging from 10 to 100 mM. The addition of organic solvents was also attempted during method development. In this case, the nature and proportion of the organic modifier are given in the text. The prepared BGE solutions were filtered using 0.44 µm nylon syringe filters (Macherey-Nagel, Düren, Germany).

### 2.6. Method Validation Parameters

Method validation was performed in accordance with ICH guidelines, encompassing the sensitivity (limit of detection—LOD; limit of quantification—LOQ), linearity, accuracy, and precision (system precision and repeatability) [43]. The method sensitivity in terms of LOD and LOQ values was evaluated at concentration values yielding a signal-to-noise ratio of approximately 3:1 and 10:1, respectively. The linearity of the method was evaluated at five concentration levels for all analytes by plotting peak areas as a function of the analyte concentration and calculating the coefficients of determination, y-intercept, and slope of the regression lines. The system precision was assessed using six replicate injections of the same analyte solution, calculating the relative standard deviation (RSD%) percentages of the migration times and peak areas for each analyte. The accuracy and precision (repeatability) were assessed at the same three concentration levels, using three replicates at each concentration level for each analyte, and evaluated in terms of the recovery percentage for accuracy and RSD percentage for repeatability.

## 3. Results

### 3.1. Method Development for the Determination of Furocoumarins

The method development focused on achieving baseline separation in the shortest possible analysis time, enabling the rapid screening of the selected furocoumarins. Thus, a high countercurrent electro-osmotic flow (EOF) velocity was generated using a high pH BGE, alongside short-end injection near the detection window, thereby reducing the effective length of the capillary to the shortest possible length (8.5 cm). To achieve selectivity, various BGE additives were tested as pseudostationary phases, including two anionic cyclodextrins (SBE-β-CD and S-β-CD) and two anionic surfactants (SDS and SDC).

**Figure 2:**
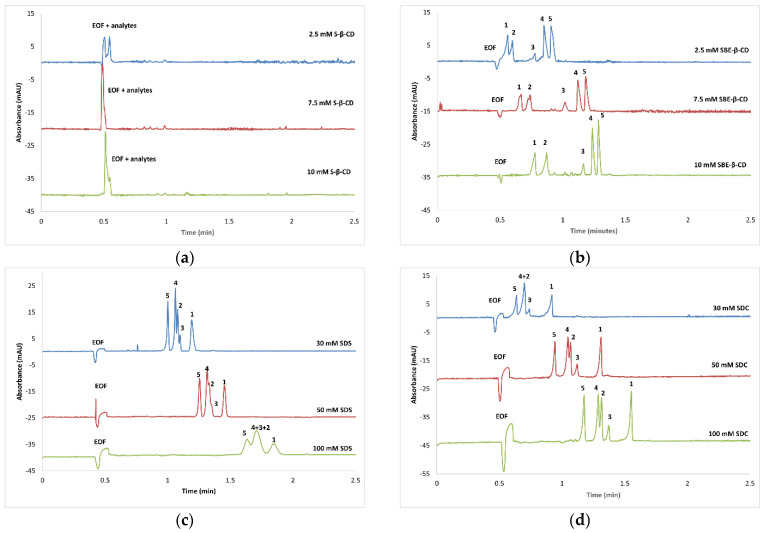
Representative electropherograms obtained during the initial development of the CE method. CE conditions: 10 mM sodium tetraborate, supplied with the indicated BGE additives: (**a**) S-β-CD, (**b**) SBE-β-CD, (**c**) SDS, (**d**) SDC. Capillary temperature: 25 °C; injection: −30 mbar × 2 s; applied voltage: −20 kV (short-end, normal polarity mode). Detection at 315 nm. Analytes: 1—imperatorin; 2—bergapten; 3—isobergapten; 4—isopimpinellin; 5—xanthotoxin.

The results obtained with increasing concentrations of the abovementioned BGE additives are shown in Figure 2. To determine the effect of adding the CDs to the BGE on the separation of the selected furocoumarins, increasing concentrations of CDs were used. Interestingly, no interactions were observed between S-β-CD and the analytes (Figure 2a); however, the use of SBE-β-CD showed promising results (Figure 2b). The migration order upon application of this CD derivative was imperatorin, isobergapten, bergapten, and isopimpinellin, followed by xanthotoxin. Based on the migration order, it can be established that xanthotoxin forms the most stable complex, while imperatorin forms the least stable one. As expected, an increasing CD concentration leads to increased migration times and increased selectivity, but also higher electric currents. Under the applied conditions, baseline resolution was achieved between all analytes; nonetheless, asymmetric peaks were obtained, as excessive peak fronting was observed for the first three peaks, while the last two peaks were characterized by peak tailing.

Further method development was performed using MEKC, employing SDS as a micelle-forming agent, in 10 mM sodium tetraborate. As can be observed from Figure 2c, increasing the SDS concentration resulted in longer analysis times; however, the method’s selectivity was not improved.

Another approach to attain simultaneous separation of the analytes was to increase the sodium tetraborate concentration of the BGE to 50 mM, which resulted in significantly improved separation, even at lower SDS concentrations. The migration order was different in this case compared to the application of SBE–β–CD, as xanthotoxin migrated first, followed by isopimpinellin, bergapten, isobergapten, and imperatorin. In this case, baseline separation of the analytes could be achieved at 30 mM SDS concentration; however, this was accompanied by relatively long migration times, especially in the case of imperatorin (migration time > 10 min).

Changing SDS to SDC in the BGE led to improved separation performance. As observed in Figure 2d, using 50 mM sodium tetraborate and 30 mM SDC, baseline resolution was achieved between all analytes, except for isopimpinellin and isobergapten. Some advantages of using SDC, instead of SDS, include shorter migration times and higher efficiency; thus, this BGE was used for further method optimization.

**Figure 3:**
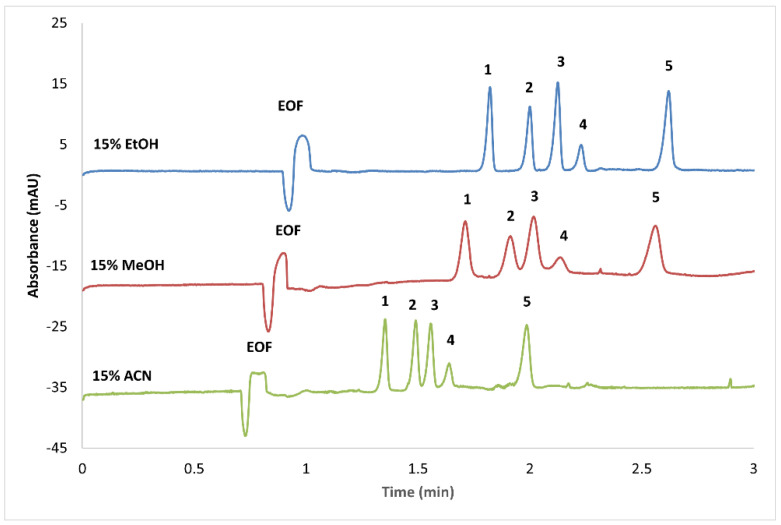
Separation of furocumarins using solvent-modified MEKC. CE conditions: 10 mM sodium tetraborate, supplied with 100 mM SDC, and the indicated organic solvents. Other conditions are the same as in Figure 2. Analytes: 1—xanthotoxin; 2—isopimpinellin; 3—bergapten; 4—isobergapten; 5—imperatorin.

To further improve the separation of the selected furocoumarins, three different organic solvents were added to the BGE at increasing proportions. For comparison, the results at 15% *v/v* organic solvents are depicted in Figure 3. As can be observed, the best selectivity in the shortest analysis time was obtained with 15% *v/v* EtOH, as in this case, all analytes were baseline-separated in under 3 min. Although a higher EtOH proportion in the BGE led to better selectivity, longer migration times and peak broadening were also observed.

**Figure 4:**
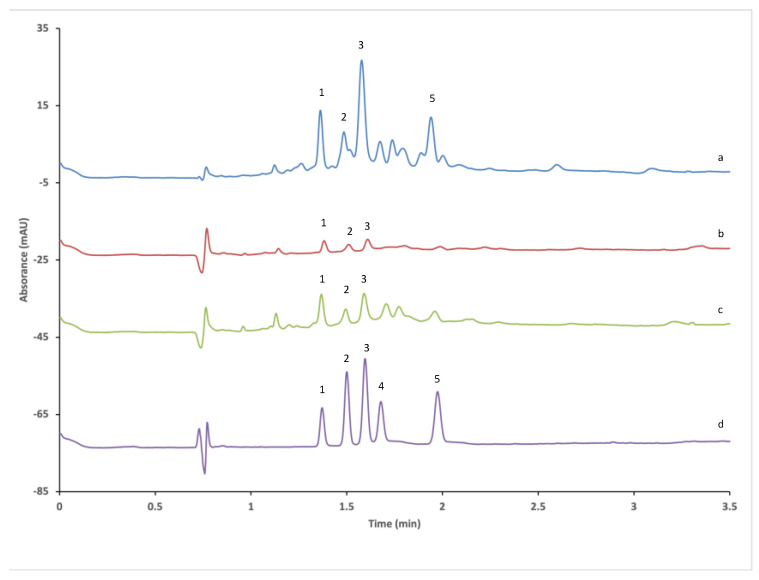
Representative electropherograms obtained during the application of the method for the analysis of food supplements. (a) Sample 8, (b) sample 9, (c) sample 10, (d) analyte solution. CE conditions: 10 mM sodium tetraborate, 100 mM SDC in 85:15 *v*/*v*% water:EtOH. Capillary temperature: 25 °C; injection: −30 mbar × 2 s; applied voltage: −30 kV (short-end, normal polarity mode). Detection at 268 nm. Analytes: 1—xanthotoxin; 2—isopimpinellin; 3—bergapten; 4—isobergapten; 5—imperatorin. Unmarked peaks are unidentified components in the samples.

A further decrease in analysis time was attained by increasing the applied voltage to 30 kV, which resulted in the simultaneous baseline separation of the selected furocoumarins in under 3 min run time using the optimized MEKC conditions: BGE consisting of 10 mM sodium tetraborate, 100 mM SDC, 15% EtOH; applied voltage of 30 kV; 25 °C capillary temperature; and short-end hydrodynamic injection with 30 mbar × 2 s (see Figure 4d).

### 3.2. Method Validation

Method validation was performed at 268 nm detection wavelength, focusing on sensitivity (determination of LODs and LOQs), linearity, accuracy, and precision (system precision and repeatability). The results of the method validation are summarized in Table 1.

The linearity of the method was assessed in the range of 2.5–100 μg/mL, at five concentration levels for all analytes. As the results indicate, the method showed an excellent correlation between concentration and peak areas for all the tested furocoumarins. The system precision was tested by injecting the same analyte solution (concentration: 50 μg/mL) six times. The obtained RSD% values were lower than or equal to 0.82% for migration times, and lower than or equal to 3.13% for peak areas. The accuracy of the method was evaluated at three concentration levels (10, 20, and 50 μg/mL for isobergapten, while the highest concentration was 100 μg/mL for the other analytes), with recoveries ranging from 93.54% to 107.30%. The intra-day precision was assessed at the same concentration levels with three replicates. The obtained RSD% values ranged from 1.28% to 4.62%.

The results indicated that the method can be applied for the determination of the selected furocoumarins.

### 3.3. Method Application

#### 3.3.1. Determination of Furocoumarins in Herbal Extracts

The developed method was successfully applied for the rapid screening of bergapten, isobergapten, isopimpinellin, imperatorin, and xanthotoxin from extracts obtained from different organs of the *Heracleum sphondylium* plant. The monitored furocoumarins were identified based on their migration time and UV spectra.

The monitored furocoumarins were detected only in root (sample 1) and fruit extracts (sample 6); none of the stem (samples 2, 3, 5) or leaf (sample 4, 7) extracts seemed to contain furocoumarins above the LOD. Table 2 summarizes the obtained furocoumarin content in ethanolic extracts of roots and fruits of *H. sphondylium*.

Since commercially available tinctures use a wide range of concentrations of EtOH, we prepared extracts with 30%, 50%, and 80% aqueous EtOH. Dresler [41] used 80% MeOH to extract furocoumarins, obtaining over 8 mg/g isopimpinellin and 5 mg/g bergapten from the *Heracleum sphondylium* herb. Out of all the plant parts tested, extracts from the root with 50% and 80% EtOH contained the highest concentrations of the monitored furocoumarins, specifically over 6 mg/g of total furocoumarins. In contrast, green fruits contained approximately half of this amount (Table 2).

#### 3.3.2. Furocoumarin Determination in Food Supplements

The developed method was also challenged by analyzing *Heracleum sphondylium*-containing food supplements obtained from local pharmacies, drug stores, and online stores. We analyzed a total of 10 food supplement samples, comprising 4 tinctures, 5 capsule formulations, and 1 uncoated tablet formulation (for details, see Supplementary Material, Table S1), to measure their furocoumarin content. Table 3 reports the amounts of furocoumarins in the food supplements that were tincture. Furocoumarins were not detected in any of the solid forms of food supplements (capsules or tablets). A selection of the obtained electropherograms is depicted in Figure 4.

Selected real samples were also analyzed using an HPLC-UV, as described in the Supplementary Material. The results obtained, as presented in Supplementary Table S2, indicate a good quantitative agreement between the two methods. As expected, the HPLC-UV method excelled in sensitivity, as observed for imperatorin, which could not be accurately determined in root extracts by the MEKC method, as it was below the LOD, but could be measured by the HPLC method. The latter method also provided higher selectivity, albeit at the cost of a significantly longer analysis time (20 min for the HPLC-UV method vs. 3 min for the MEKC method, excluding re-equilibration time and preconditioning), as well as higher costs. A comprehensive comparative study is currently in preparation and will be reported separately.

## 4. Discussion

### 4.1. Development of an Analytical Method for the Determination of Furocoumarins

The selected furocoumarins are neutral under the typical pH range that can be used in CE. Several approaches have been successfully implemented to induce selectivity and facilitate the separation of neutral compounds in electromigration-based techniques. These include the use of stationary (capillary electrochromatography) or pseudostationary phases (electrokinetic chromatography, EKC). The latter approach is particularly favored due to its ease of use, its flexibility, and the absence of specialized instrumentation. Frequently employed pseudostationary phases include surfactants above their critical micellar concentration (micellar electrokinetic chromatography, MEKC), microemulsions (microemulsion electrokinetic chromatography, MEEKC), and CDs (CD-EKC).

In most cases, where electromigration-based methods were used for the determination of coumarins, alkaline BGEs were employed, typically at a pH of 9–10; however, in some cases, somewhat lower pH values were also attempted. In all the examined cases, the use of pseudostationary phases was necessary, either through the use of MEKC or by adding anionic surfactants or CDs to the BGE to confer anodic mobility to the analytes. The use of MEKC is widely applied in phytochemical analysis, where, in numerous cases, neutral compounds need to be separated.

In this study, two widely used anionic CDs, S-β-CD and SBE-β-CD, were first employed as pseudostationary phases. Subsequently, the anionic surfactants SDS and SDC were utilized as micelle-forming agents to facilitate the separation of the targeted analytes. Due to the anionic character of the selected BGE additives, upon interaction with the analytes, all furocoumarins displayed anionic mobility, migrating after the EOF.

As can be observed, in addition to S-β-CD, all the other BGE additives displayed interactions with the analytes (Figure 2). Although the use of SBE-β-CD showed promising results, unfortunately, higher concentrations could not be used due to the increased currents generated, which led to system instabilities. In general, the most promising results, with the shortest analysis time and acceptable peak shapes, were achieved when using SDC as the pseudostationary phase. However, using SDC alone did not offer baseline separation for all furocoumarins, as isopimpinellin and isobergapten co-migrated. To further enhance the method selectivity, the addition of organic modifiers was also tested. Adequate selectivity and better peak shapes were observed with the use of EtOH. Subsequent optimization involved fine-tuning the applied voltage and capillary temperature parameters, resulting in the successful separation of the targeted analytes and establishing the final analytical method.

### 4.2. Determination of Furocoumarins

The ultrasound-assisted extraction method employed in our study is the most commonly used method for obtaining furocoumarins from various foods and plant products [44]. Over the years, the most suitable extraction solvents have been found to be MeOH, EtOH, and ethyl acetate [44].

Based on the study performed by Kerekes on the extractability of certain furocoumarins [45], bergapten is best extracted in chloroform, acetone, and MeOH, while imperatorin is best extracted in ACN, toluene, EtOH, and ethyl acetate. Although, according to this study, it would have been ideal to extract the furocoumarins with acetonitrile, given that many commercialized food supplements are hydroethanolic extracts (tinctures), we decided to use EtOH as the extraction solvent. In terms of EtOH concentration, commercial tinctures display significant variety (see Table S1/Supplementary material); consequently, the use of 30%, 50% and 80% EtOH was decided upon for the extraction of furocoumarins.

Furocoumarins in edible plants are mainly found in species of the Rutaceae and Apiaceae families [2]. The 6.25 mg/g total furocoumarin content of *Heracleum sphondylium* root may be considered relatively high in foods [1,46,47], but is also considered in herbal medicines. For example, levels of over 4.55 mg/g have recently been reported for *Angelica* root in extracts obtained using 80:20 MeOH/water mixture [46]. However, the high furocoumarin content of the plant product does not necessarily mean that the preparations made from it will also have high furocoumarin contents. For example, infusions have been shown to extract about half of the total furocoumarins from *Angelica* root [46].

In the case of the *Heracleum sphondylium* root, in extracts obtained with 50% EtOH, the main compound is isopimpinellin, the amount of which is twice that of isobergapten and bergapten. In the fruit, the main compound is imperatorin, which was not detected in root samples.

Most toxicological studies on furocoumarins focus on xanthotoxin and bergapten; xanthotoxin (8-methoxypsoralen) is believed to have the most significant degree of photosensitizing activity and is therefore the main psoralen used in PUVA [2]. In the roots and fruits of *Heracleum sphondylium*, xanthotoxin is present in relatively small amounts; its extractability is better with 50% EtOH than with 30% EtOH. *Heracleum mantegazzianum* Sommier et Levierm is considered toxicologically the most dangerous species of the genus, containing much higher amounts of furocoumarins; the fruits contain the maximum amount (396 mg/100 g fresh weight), followed by the leaves (256 mg/100 g fresh weight), while minimum amounts are found in the stem [48]. Angelicin, 8-methoxypsoralen, and psoralen are the major compounds in the leaves, and bergapten is in the fruit of *H. mantegazzianum* [48,49].

The developed and validated MEKC method was used to determine the furocoumarin content in food supplements labeled as containing *Heracleum sphondylium*. The product label typically specifies the part of the plant that was used to obtain it, provides suggestions for use, and outlines the health benefits and possible contraindications of the food supplement (see Table S1/Supplementary material). As mentioned in the results, no furocoumarins were detected in any of the capsule or tablet food supplements; however, significant amounts were found in the tinctures.

To assess the extent of furocoumarin ingestion resulting from consuming these food supplements, maximum daily intake estimates were calculated. These estimates were derived from a combination of our experimental results and the product label information. According to the manufacturer of the tincture-type food supplements, it is recommended to take one tablespoon of tincture three times a day. To calculate the amount of furocoumarin that is ingested with a single dose and over a day, the following formulas were employed (1 tablespoon corresponds to 15 mL):

Estimated intake per one dose (tablespoon) = [(furocoumarin mg/mL) × 15]

Estimated maximum daily dose = [(furocoumarin mg/mL) × 15 × recomended number of tablespoons/day]

As illustrated in Table 4, the highest amount of furocoumarins is achieved through the ingestion of food supplement sample 8, with levels reaching up to 19.97 mg/day. Conversely, the lowest intake is observed in sample 9, which contains less than 5 times that of sample 8.

The observed variations in the composition of the tincture samples can be attributed to the utilization of distinct plant parts during the production process. According to the information provided on the packaging, the tincture with the highest furocoumarin content (sample 8) is obtained from the root and seeds of *Heracleum sphondylium*. Notably, sample 8 also contains imperatorin, a substance that was exclusively detected in the fruit samples. The absence of isobergapten in the tinctures is likely attributable to the reduced extractability of this substance in the lower EtOH concentration employed during their preparation.

As stated in the results section, furocoumarins were not detected in the stem and leaf samples from *Heracleum sphondylium*. Aerial-type plant products are typically harvested during the flowering stage. In the case of *Heracleum sphondylium* and other Apiaceae plants, this is particularly relevant due to their protracted flowering period, which can result in the presence of green fruits in these plant products. Consequently, the presence of imperatorin, a compound that is characteristic of the green fruits of *Heracleum sphondylium*, was anticipated in certain food supplements. However, except for sample 10, imperatorin was not detected above the LOD in any of the samples examined.

According to the Scientific guidelines of the European Medicine Agency (EMA) [27], exposure through food to amounts of furocoumarins below 1.5 mg per day is considered harmless. From this point of view, three of the four tinctures we have tested have a higher intake of furocoumarin than this, up to 13 times the harmless limit. In two of the samples analyzed, the dose that is considered harmless is exceeded, already at a single dose (samples 8 and 10). However, the consumption of 200 g of parsnips or celery, with an average furocoumarin content of approximately 20–50 mg/kg, results in an estimated intake of 4–10 mg per person [50]. Higher intakes should be expected when consuming parsley, which can contain up to 137 mg/kg of total furocoumarin [46].

The lowest dose of furocoumarin that has shown visible phototoxic effects in adults, in combination with UVA, is 14 mg of xanthotoxin or the combination of 10 mg of xanthotoxin with 10 mg bergapten [50,51]. According to estimates, the population in Western countries consumes furocoumarins two to three orders of magnitude below the lowest doses described as toxic in animal experiments at subchronic and chronic administration [50].

## 5. Conclusions

This study presents the development and analytical performance verification of a rapid and cost-effective MEKC method for determining furocoumarins in *Heracleum sphondylium* extracts and food supplements that contain *Heracleum sphondylium*. The experimental procedure involves a simple ultrasound extraction method, followed by an ultrafast determination of furocoumarins by MEKC, using a BGE composed of 10 mM sodium tetraborate, 100 mM SDC in water:EtOH 85:15 *v*/*v*%, employing hydrodynamic short-end injection at 30 mbar × 2 s on a bubble cell capillary (effective capillary length = 8.5 cm, total length = 38.5 cm, bubble factor = 3), thermostated at 25 °C and using −30 kV applied voltage and hydrodynamic sample injection.

The analytical performance of the method was evaluated in terms of the sensitivity, linearity, accuracy, and precision (system precision and repeatability), and the method was deemed suitable for analyzing the selected furocoumarins.

The analytical performance of the method, particularly its short analysis time and low cost, supports its potential applicability for the routine screening of the five furocoumarins in the control of herbal products and food supplements. Overall, the results demonstrated that the CE can be applied as an alternative technique to the analysis of furocoumarins in herbal products and food supplements.

Overall, out of the ten food supplements analyzed, in seven products no furocoumarins above the LOD were detected; in one product the amount was close to the dose that is considered harmless, and in two the amount was well above the dose that is considered harmless.

## Figures and Tables

**Table 1:** Summary of results obtained during method performance verification.

Parameter	Level	Xanthotoxin	Isopimpinellin	Bergapten	Isobergapten	Imperatorin
Range (μg/mL)		5–100	2.5–100	2.5–100	2.5–50	2.5–100
Regression equation		y = 0.7098x−0.3895	y = 1.2534x + 1.903	y = 1.5857x + 0.9365	y = 1.3935x + 3.3618	y = 1.2153x + 4.7527
r^2^		0.996	0.99	0.993	0.999	0.999
LOD (μg/mL)		2	1	1	1	1
LOQ (μg/mL)		5	2.5	2.5	2.5	2.5
Accuracy (recovery%)	10 μg/mL	105.60%	102.15%	99.87%	102.02%	99.40%
	20 μg/mL	93.54%	101.37%	102.69%	106.99%	104.09%
	50/100 μg/mL *	104.30%	95.54%	98.21%	101.10%	103.65%
System precision (RSD%)	20 μg/mL					
Migration time		0.29%	0.12%	0.45%	0.54%	0.82%
Peak area		3.13%	1.66%	2.15%	2.79%	2.43%
Repeatability (RSD%)	10 μg/mL	4.22%	3.69%	4.62%	4.43%	4.57%
	20 μg/mL	2.52%	2.69%	2.29%	3.16%	2.55%
	50/100 μg/mL *	1.40%	2.70%	2.25%	2.51%	1.28%

* 50 μg/mL for isobergapten; 100 μg/mL for the other analytes.

**Table 2:** Content of furocoumarins in the ethanol extracts of *Heracleum sphondylium* (in mg/g).

Sample Type, EtOH Concentration Used for Extraction (%)	Root, 30%	Root, 50%	Root, 80%	Green Fruits, 30%	Green Fruits, 50%	Green Fruits, 80%
**Compound**						
Xanthotoxin	0.23 ± 0.006	0.35 ± 0.002	0.51 ± 0.073	0.34 ± 0.02	0.49 ± 0.032	0.61 ± 0.037
Isopimpinellin	0.38 ± 0.102	3.09 ± 0.128	2.94 ± 0.105	0.20 ± 0.002	0.31 ± 0.021	0.38 ± 0.010
Bergapten	0.17 ± 0.002	1.28 ± 0.122	1.80 ± 0.043	0.52 ± 0.014	0.87 ± 0.101	0.86 ± 0.103
Isobergapten	0.24 ± 0.003	1.50 ± 0.143	1.00 ± 0.057	<LOD	<LOD	<LOD
Imperatorin	<LOD	<LOD	<LOD	0.30 ± 0.032	1.01 ± 0.062	1.67± 0.072
TOTAL	1.02 ± 0.113	6.23 ± 0.454	6.25 ± 0.278	1.37 ± 0.068	2.68 ± 0.217	3.52 ± 0.222

LOD—limit of detection.

**Table 3:** Concentration of furocoumarins in tincture-type food supplements (in μg/mL).

Compound	Sample 8	Sample 9	Sample 10
Xanthotoxin	153.83 ± 6.31	35.07 ± 3.82	82.94 ± 4.48
Isopimpinellin	57.61 ± 2.20	12.35 ± 1.34	23.28 ± 0.99
Bergapten	143.23 ± 10.72	28.11 ± 1.92	39.12 ± 1.58
Isobergapten	<LOD	<LOD	<LOD
Imperatorin	89.04 ± 9.51	<LOQ	24.10 ± 1.57
TOTAL	443.71 ± 28.74	75.53 ± 7.08	169.44 ± 8.62

**Table 4:** Estimated daily intakes of furocoumarins from food supplements.

Compound	Sample 8		Sample 9		Sample 10	
	mg/tbsp	mg/day	mg/tbsp	mg/day	mg/tbps	mg/day
Xanthotoxin	2.31	6.92	0.53	1.58	1.24	3.73
Isopimpinellin	0.86	2.59	0.19	0.56	0.35	1.05
Bergapten	2.15	6.45	0.42	1.26	0.59	1.76
Isobergapten	<LOD	<LOD	<LOD	<LOD	<LOD	<LOD
Imperatorin	1.34	4.01	<LOD	<LOD	0.36	1.08
TOTAL	6.66	19.97	1.13	3.40	2.54	7.62

tbsp—tablespoon; LOD—limit of detection.

## Data Availability

The original contributions presented in the study are included in the article/Supplementary Material, further inquiries can be directed to the corresponding author.

## Supplementary Materials

The following supporting information can be downloaded at https://www.mdpi.com/article/10.3390/foods14132348/s1: Table S1: *Heracleum sphondylium*-containing food supplements: product name, producer, declared composition, suggested health benefits, contraindications, lot, expiry dates; Table S2. Quantitative comparison of furocoumarins in *Heracleum sphondylium* root extracts measured by CE and HPLC (µg/mL ± SD).

## Abbreviations

The following abbreviations are used in this manuscript:

ACN	Acetonitrile
BGE	Background electrolyte
CD	Cyclodextrin
CZE	Capillary zone electrophoresis
CH_2_Cl_2_	Dichloromethane
EFSA	European Food Safety Authority
EKC	Electrokinetic chromatography
EtOH	Ethanol
EOF	Electro-osmotic flow
HPLC-UV	High-performance liquid chromatography with ultraviolet detection
MEEKC	Microemulsion electrokinetic chromatography
MeOH	Methanol
MEKC	Micellar electrokinetic chromatography
LC-MS	Liquid chromatography–mass spectrometry
LOD	Limit of detection
LOQ	Limit of quantification
RSD	Relative standard deviation
S-β-CD	Sulfated-β-cyclodextrin
SBE-β-CD	Sulfobutylated-β-cyclodextrin
SDC	Sodium deoxycholate
SDS	Sodium dodecyl sulfate
tbsp	Tablespoon
