# Supplementary material for: Fast Determination of Furocoumarins in Food Supplements Containing Heracleum sphondylium L. Using Capillary Electrophoresis
Source: Foods. 2025 Jul 2;14(13):2348. doi: 10.3390/foods14132348 (PMC12249021; doi:10.3390/foods14132348)
Supplement: Supplementary file 1 [file foods-14-02348-s001.zip › foods-3661877-supplementary.pdf]

SUPPLEMENTARY MATERIAL

Fast Determination of Furocoumarins in Food Supplements Containing *Heracleum sphondylium* L. Using Capillary Electrophoresis

Eszter Laczkó Zöld [1], Csenge Kis [2] , Erzsébet Nagy-György [1], Erzsébet Domokos [3], Elek Ferencz [4] and Zoltán-István Szabó [5,6,*]

[1] Department of Pharmacognosy and Phytotherapy, “George Emil Palade” University of Medicine, Pharmacy, Science, and Technology of Targu Mures, 540139 Târgu Mureș, Romania; eszter.laczko@umfst.ro (E.L.Z.); gyorgybobe@gmail.com (E.N.-G.)

[2] Department of Physical Chemistry, “George Emil Palade” University of Medicine, Pharmacy, Science, and Technology of Targu Mures, 540139 Târgu Mureș, Romania; csenge.kis@umfst.ro

[3] Faculty of Technical and Human Sciences, Sapientia Hungarian University of Transylvania, 540485 Târgu Mureș, Romania; domokos.erzsebet@ms.sapientia.ro

[4] Legal Medicine Service, Emergency County Hospital Miercurea Ciuc, 530173 Miercurea Ciuc, Romania; elekferencz@yahoo.com

[5] Department of Industrial Pharmacy and Pharmaceutical Management, “George Emil Palade” University of Medicine, Pharmacy, Science, and Technology of Targu Mures, 540139 Târgu Mureș, Romania

[6] Sz-Imfidum Ltd., 525401 Lunga, Romania

* Correspondence: zoltan.szabo@umfst.ro

Table S1. ***Heracleum sphondylium*** **containing food supplements: products name, producer, declared composition, suggested health benefits and contraindications (according to label), lot, expiry dates**

Sample number	Brand name	Producer, location,	Home page	Dose form	Declared composition	Suggested use	Suggested effect, health benefits and indications	Contraindications, precautions	Lot	Expiry date
**Sample8**	Brânca ursului	Dacia Plant SRL, Bod – 507015, str. Hărmanului. Jud. Brașov, Romania	https://www.daciaplant.ro/branca-ursului-tinctura.html	50 ml tincture	Root and seeds of Heracleum sphondylium	3x1 tablespoon, 30 minutes before meals	- supports sexual health and reproductive function,<br>- contributes to a normal sex life,<br>- contributes to maintaining comfort during menopause,<br>- protects sperm and supports sex drive,<br>- Aphrodisiac.	Benign prostate hypertrophy (prostate adenoma), uterine fibroids, genital cancers, pregnancy, breastfeeding, intolerance to any of the components of the product.	5254	30.06.2026
**Sample 9**	Brânca ursului	SC Dorel Plant SRL, 417121, Poienii de Jos 15, jud. Bihor, Romania	https://dorelplant.ro/product/tinctura-de-branca-ursului/	200 ml, tincture	20% roots of Heracleum sphondylium	3x1 tablespoon, before meals	This product is a stimulant of gland activity, aphrodisiac, regulator of nervous activity, stimulant of non-specific local immunity (renal and genital), general tonic (digestive, nervous), genital vasodilator, impotence, frigidity, sterility in women and men, premature menopause, ovarian failure.	Do not exceed the recommended dose for daily consumption. Do not take with antibiotics.	01	06.06.2026
**Sample 10**	Brânca ursului	Faunus Plant SRL,	https://faunu	200 ml, tincture	20% aerial parts of	3 tablespoons	Normalization of the secretory activity of the	Children under 12 years old, pregnancy,	8	08.2026

		Poienii de Jos nr 25, Jud. Bihor, Romania	splant.ro/products/tinctura-branca-ursului?_pos=2&_psq=branca&_ss=e&_v=1.0		Heracleum sphondylium	of tincture per day, one tablespoon 30 minutes before main meals	male and female sex glands<br>Improved tone<br>Reduction of fatigue	breastfeeding, allergy to hogweed, people who are prohibited/limited in alcohol consumption.		
**Sample 11**	Tinctura de Brânca ursului	Aroma Plant Bonchis SRL, Bunești nr 83, jud. Bihor, Romania	https://aromaplantbonchis.ro/produse/tinctura-branca-ursului	500 ml tincture	still water 50%; hogweed – aerial parts (Heracleum sphondylium) 27 %; alcohol (96%) - 23%	3 tablespoons of tincture per day, one tablespoon 30 minutes before main meals	Reduction of fatigue;<br>- Improving the general condition and resistance of the body;<br>- Optimization of sperm count;<br>- Increased libido and vitality of the body in both men and women;<br>- Supports the secretory activity of the sex glands in both sexes;	Prostate adenoma, uterine fibroids, pregnancy, genital cancers, intolerance to any of the components of the product, people who are strictly forbidden to consume alcohol	5	01.09.2028
**Sample 12**	Brânca ursului extract	Rotta Natura SA, București, str.Intr. Grigore Alexandrescu nr 6/1, sector 1	https://www.rottanatura.com/cauta/branca%20ursului	60 capsules	5:1 extract from hogweed root (Heracleum sphondylium) - 400 mg	One capsule, 3 times a day, 20-30 min. before meals. Up to 6 capsules per day can be consumed.	stimulates the activity of the sex glands<br>maintains sexual health<br>helps eliminate the discomfort caused by menopause<br>vasodilatator the genital level	Contraindicated for men with prostate adenoma, genital cancer	25	10.2025

**Sample 13**	Brânca ursului	Dacia Plant SRL,		60 tablets	Root extract, powder from aerial part and root	Adults: 1 tablet per day. In exceptional cases, 2-4 tablets can be administered once 30 minutes before sexual intercourse.<br><br>3-month courses with a 3-week break are recommended.	- supports fertility in both sexes;<br>- potent mints;<br>- contributes to a good sex life<br>-aphrodisiac.	Pregnancy, breastfeeding, intolerance to any of the components	BRN0723	31.07.2026
**Sample 14**	Brânca ursului	Laboratoarele Favisan, Lugoj, str. C.D.Loga 36, jud. Timiș, Romania	https://www.favisan.ro/produs/branca-ursului-capsule/	40 capsules	Heracleum herba – 200 mg	3-6 capsules per day	adjuvant in male and female infertility, venereal diseases, adnexitis, metroanexitis, renal failure, hypertension	-	02/29.09.23	07.2026
**Sample 15**	Brânca ursului	Pro Natura, SC Laboratoarele Medica SRL,	https://pro-natura.ro/shop/bran	60 capsules	250 mg hogweed powder (not specified	1cps x 3/day, before meals by 20min.	The hogweed, nicknamed "Romanian ginseng", has strong aphrodisiac properties	-	1827	09.2026

		Otopeni, str. Frasinului 11	ca-ursului-x-60-capsule/		which plant part)		and is aimed at both women and men.<br>For regulation of hormonal balance			
**Sample 16**	Brânca marelui urs	Herbagetica SRL, Brașov, Hărman, Podu Oltului, Str. Florilor 1085	https://herbagetica.ro/branca-marelui-urs233.html?___SID=S	60 capsules	10:1 extract of hogweed (Heracleum sphondylium) - 175.00 mg; Nettle (Urtica dioica) powder - 28.00 mg; 4:1 extract of Tribulus terrestris L. standardized with 40% saponins - 119.00 mg; Chinese Ginseng (Panax ginseng) root powder - 28,00 mg	2 capsules 3 times a day (in courses of maximum 2 weeks)	vascular, hormonal, mental impotence<br>Infertility in men<br>premature andropause<br>premature aging<br>male hormone deficiency<br>low libido	-	5570	13.10.2026
**Sample 17**	Brânca ursului 4000	Cosmo Pharm SRL, București, Bd. Corneliu Coposu nr 5, Bl 103, ap 74	https://www.cosmopharm.eu/produs/branca-ursului-4000/	30 capsules	Root extract Standardized 10:1 extract from hogweed roots – 400 mg	1-2 capsules per day, taken before meals	lack of sex drive;<br>male and female sterility;<br>vascular impotent;<br>hormonal impotence;<br>genital boluses;<br>fatigue, exhaustion;<br>stress, anxiety;<br>cold hands and feet;	Specified only on homepage:<br>Hogweed is not recommended for people who have low blood pressure, those who suffer from prostate diseases, as well as pregnant or	BU20230222	02.2026

							Hypertension	breastfeeding women.<br>It causes photosensitivity. It is recommended to limit sun exposure in the hot season.		

Table S2. Quantitative comparison of furocoumarins in *Heracleum sphondylium* root extracts measured by CE and HPLC (µg/mL ± SD)

Solvent used for extraction	Ethanol 30%		Ethanol 50%		Ethanol 80%	
**Compound**	**CE**	**HPLC**	**CE**	**HPLC**	**CE**	**HPLC**
Xanthotoxin	23.19 ± 0.62	21.33 ± 0.87	34.86 ± 0.21	36.41 ± 2.58	50.85 ± 7.30	48.83 ± 5.11
Isopimpinellin	38.03 ± 10.23	40.52 ± 0.07	309.46 ± 18.77	310.86 ± 39.60	294.10 ± 10.50	300.96 ± 25.01
Bergapten	16.62 ± 0.19	19.98 ± 0.36	128.15 ± 12.18	138.03 ± 7.17	180.05 ± 4.33	186.15 ± 12.99
Isobergapten	24.32 ± 0.28	24.30 ± 0.65	150.26 ± 14.28	157.79 ± 6.44	99.70 ± 5.70	108.79 ± 2.96
Imperatorin	<LOD	1.17 ± 0.37	<LOD	2.34 ± 0.18	<LOD	4.30 ± 0.39
**TOTAL**	102.16 ± 11.32	107.30 ± 2.32	622.73 ± 45.44	645.43 ± 55.97	624.70 ± 27.83	649.03 ± 46.46

HPLC-UV method for the comparative determination of furocoumarins

HPLC-UV analyses were performed on a Thermo Finnigan Surveyor HPLC, consisting of a quaternary pump, autosampler, column thermostat, and a diode-array detector (DAD). The analytical column used for the determinations was a Purospher STAR RP-18e (150 × 4.6 mm, five µm), thermostated at 30 °C. The mobile phase consisted of 0.1% (v/v) ortho-phosphoric acid in water (A) and methanol (B). A linear gradient was employed from 25 % B to 90% B, in 20 minutes, followed by a 5-minute isocratic hold, and a 10-minute reequilibration time. The flow rate was 1 mL/min, and the injection volume was 10 µL. UV detection was performed at 220 nm.
